# Supplementary material for: The mitochondrial deoxyguanosine kinase is required for cancer cell stemness in lung adenocarcinoma
Source: EMBO Mol Med. 2019 Oct 21;11(12):e10849. doi: 10.15252/emmm.201910849 (PMC6895611; doi:10.15252/emmm.201910849)
Supplement: Supplementary file 1 — Appendix [file EMMM-11-e10849-s001.pdf]

Appendix

Appendix Figure S1

Appendix Figure S2

Appendix Figure S3

Appendix Table S1

Appendix Table S2

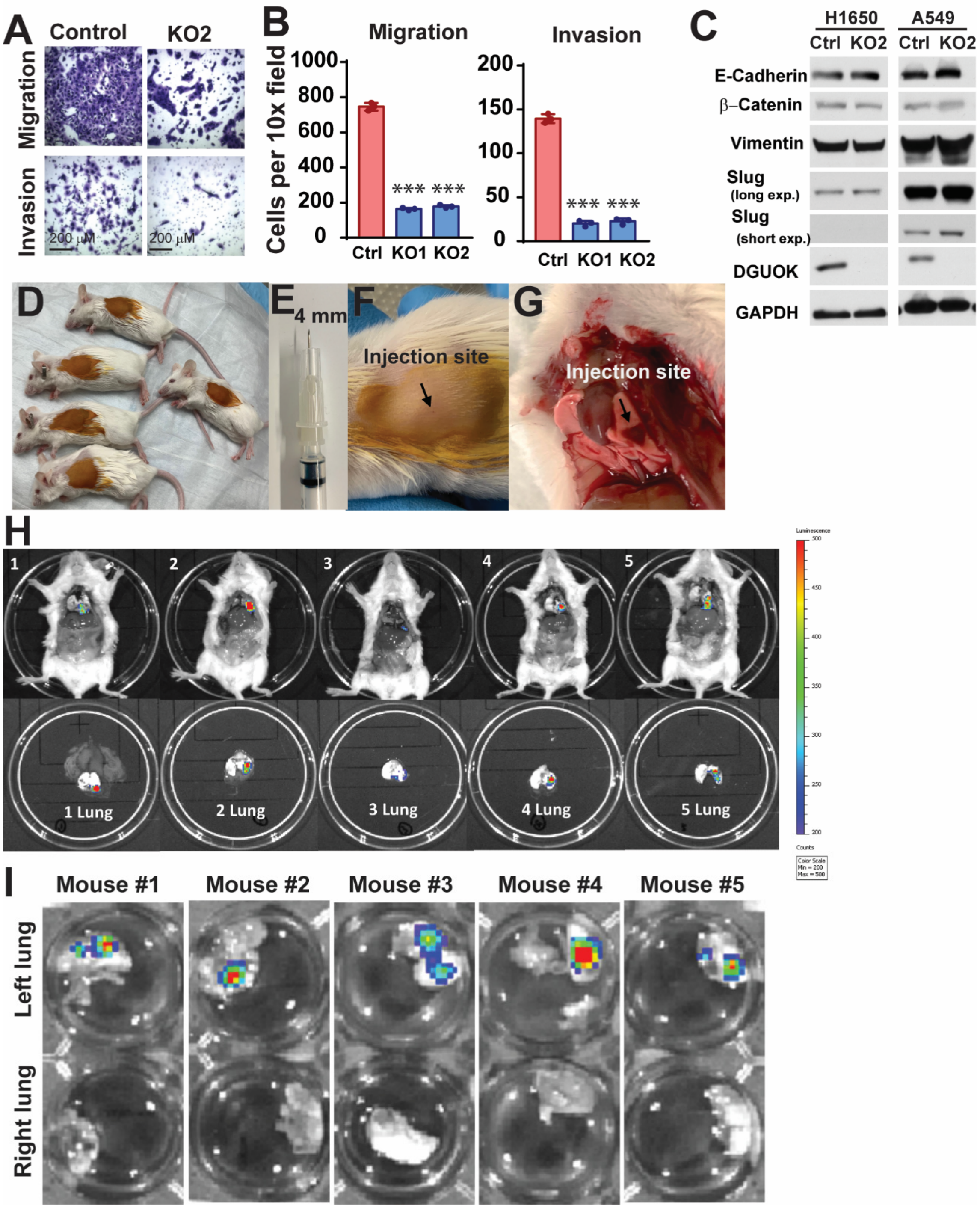

Appendix Figure S1, **A and B**, representative images (A) and quantitation (B) of cell migration /invasion assay the effect of DGUOK KO on H1650 cell migration and invasion. Data shown in **B** are from 3 technical replicates. Experiments have been repeated at least 3 times using different biological replicates with similar results. **C,** the expression levels of EMT markers in control and DGUOK KO H1650 and A549 cells. **D-I,** a pilot orthotopic injection experiment. **D,** 5 anesthetized mice before injection. **E,** an adaptor is used to ensure that the needle would not

penetrate more than 4 mm during the injection. **F,** One-ml tuberculin syringes (Becton Dickinson) with 30-gauge hypodermic needles were used to inject the cell inoculum percutaneously into the left lateral thorax, at the lateral dorsal axillary line, approximately1.5 cm above the lower rib line just below the inferior border of the scapula (arrow). **G,** mice were euthanized immediately after injection to inspect of injection sites (arrow). **H,** *ex vivo* bioluminescence imaging in the whole animal (upper panel) and extracted lung (lower panel) showing injected lung cancer cells on the left side of the lung. **I,** the left and right side of the lung were separated and subjected to *ex vivo* bioluminescence imaging. The imaging results indicate that injected lung cancer cells were only detected on the left side, but not on the right side of the lung.

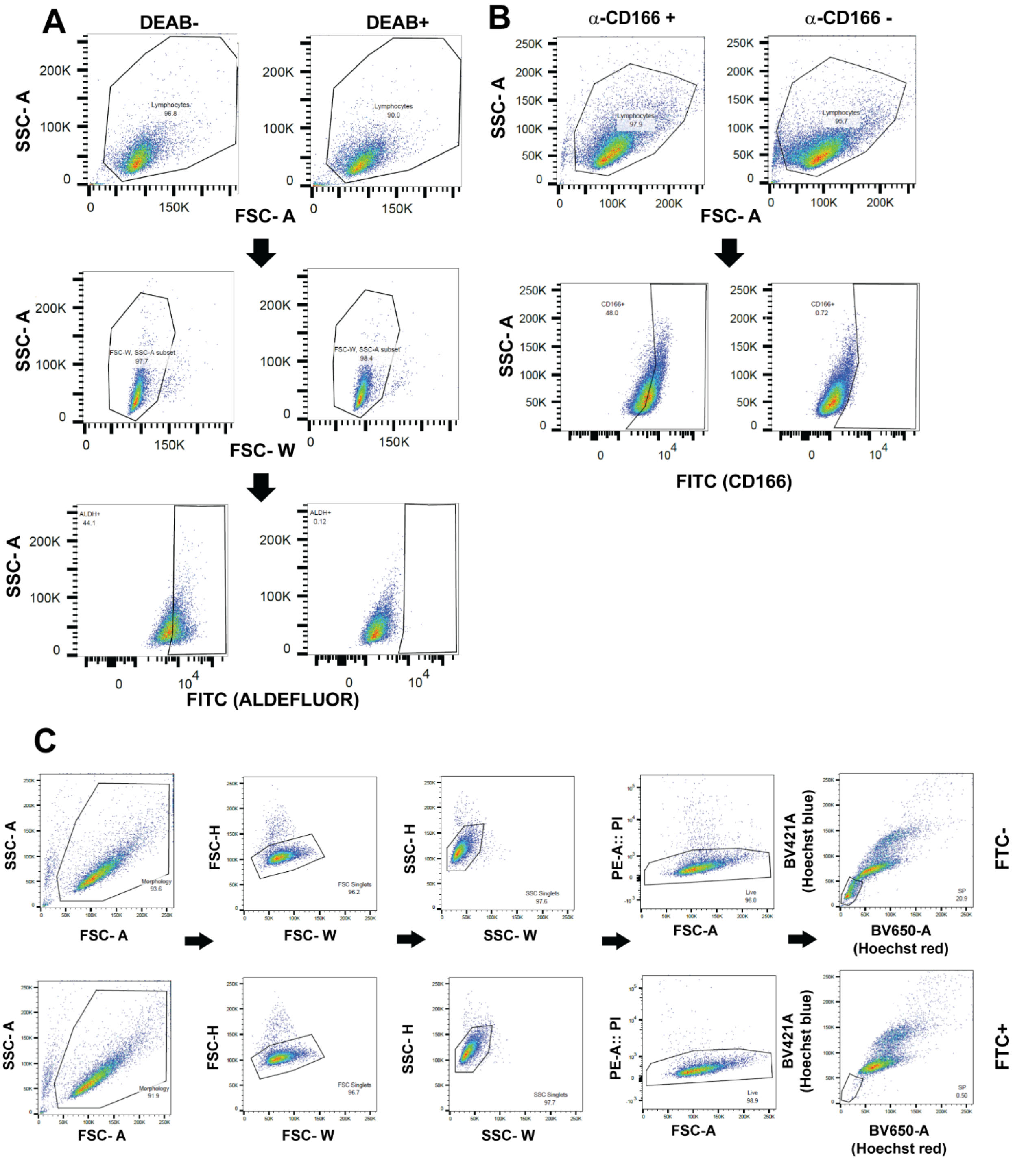

Appendix Figure S2. **A-C,** gating protocol for ALDEFLOR assay (A), CD166 staining (B) and side-population assay. DEAB and FTC treated cells were used as negative control in A and C, respectively.

Appendix Fig S3

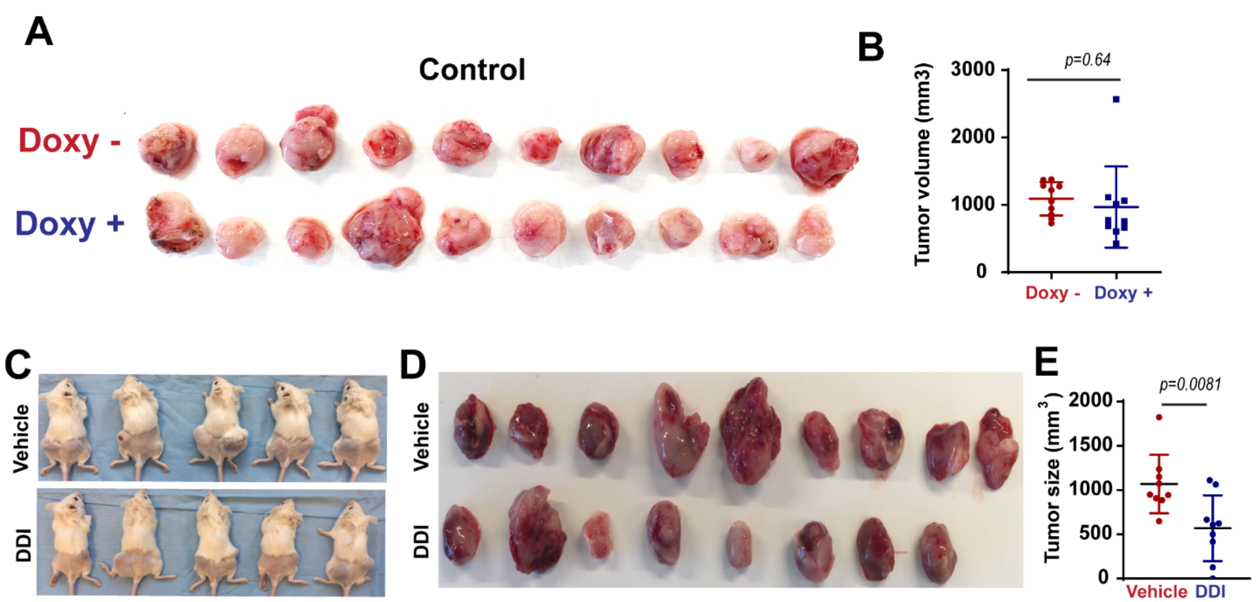

Appendix Figure S3. **A,** image of LLC allograft tumors (without DGUOK sgRNA) harvested from control mice on regular chow (Doxy -) or doxycycline chow (Doxy +). **B,** Quantitation of tumor volume for allograft tumors in A. **C-E,** images of control or DDI-treated tumor –bearing mice (**C**), resected tumor (**D**), and measurement of tumor size (**E**) at the time of euthanasia, (n=5 inoculations per group into both flank of female Albino BL6 mice).

Appendix Table S1: Oligonucleotides used for qPCR

hβACTIN-QPCR-F:<br><br>ACCAACTGGGACGACATGGAGAAA
hβACTIN-QPCR-R:<br><br>TAGCACAGCCTGGATAGCAACGTA
hCYR61_QPCR_F:<br><br>AAGAAACCCGGATTTGTGAG

hCYR61_qPCR_R:<br><br>GCTGCATTTCTTGCCCTTT
hCTGF_qPCR_F:<br><br>CCTGCAGGCTAGAGAAGCAG
hCTGF_qPCR_R:<br><br>TGGAGATTTTGGGAGTACGG
yeastNDI1_qPCR_F:<br><br>GGTGGGCCTACTGGTGTAGA
yeastNDI1_qPCR_R:<br><br>CAATGGCGAAAATGTTGTTG
hNDUFB8_qPCR_F:<br><br>CCGCCAAGAAGTATAATATGCGT
hNDUFB8_qPCR_R:<br><br>TATCCACACGGTTCCTGTTGT
hmt-ND1_qPCR_F:<br><br>TCAAACTCAAACTACGCCCTG
hmt-ND1_qPCR_R:<br><br>GTTGTGATAAGGGTGGAGAGG
hATPase6_qPCR_F:<br><br>AACCATTAACCTTCCCTCTACAC
hATPase6_qPCR_R:<br><br>GAAGTGTGAAAACGTAGGCTTG
h16s rRNA_qPCR_F:<br><br>GGGACCTGTATGAATGGCTC
h16s rRNA_qPCR_R:<br><br>TCTCGTCTTGCTGTGTTATGC

Appendix Table S2: Primers used for the quantitation of human and murine mtDNA copy numbers.

For human mtDNA quantitation

hmtDNA_MinArc_qPCR_F:CTAAATAGCCCACACGTTCCC
hmtDNA_MinArc_qPCR_R:AGAGCTCCCGTGAGTGGTTA
hmtDNA_MajArc_qPCR_F:CTGTTCCCCAACCTTTTCCT
hmtDNA_MajArc_qPCR_R:CCATGATTGTGAGGGGTAGG
hnucDNA_B2M_qPCR _F:GCTGGGTAGCTCTAAACAATGTATTCA
hnucDNA_B2M_qPCR _R:CCATGTACTAACAAATGTCTAAAATGGT

For murine mtDNA quantitation.

mmtDNA_Dloop1_qPCR_F: AATCTACCATCCTCCGTGAAACC
mmtDNA_Dloop1_ qPCR_R: TCAGTTTAGCTACCCCCAAGTTTAA
mmtDNA_Dloop2_qPCR _F: TCCTCCGTGAAACCAACAA
mmtDNA_Dloop2_qPCR _R: AGCGAGAAGAGGGGCATT
mnucDNA_Tert_qPCR _F: CTAGCTCATGTGTCAAGACCCTCTT
mnucDNA_Tert_qPCR _R: GCCAGCACGTTTCTCTCGTT
