# Supplementary material for: The mitochondrial deoxyguanosine kinase is required for cancer cell stemness in lung adenocarcinoma
Source: EMBO Mol Med. 2019 Oct 21;11(12):e10849. doi: 10.15252/emmm.201910849 (PMC6895611; doi:10.15252/emmm.201910849)
Supplement: Supplementary file 2 — Expanded View Figures PDF [file EMMM-11-e10849-s002.pdf]

Expanded View Figures

Figure EV1. DGUOK overexpression in lung adenocarcinoma.

A Kaplan–Meier analysis of the correlation of mRNA expression levels of *DGUOK* and *TK2* with overall survival in lung adenocarcinoma (Adeno.) and lung squamous cell carcinoma (Squamous) patients from Kmplot.com.
B Representative image showing the IHC staining of DGUOK in formalin-fixed, paraffin-embedded control and DGUOK KO H1650 cells.
C Representative IHC staining of DGUOK expression levels in lung adenocarcinoma specimens and paired para-tumor lung tissues.
D Western blotting showing the efficacies of DGUOK protein depletion by different sgRNAs targeting *DGUOK*. Human sg3 (KO1) and sg5 (KO2) and murine sg4 (KO1) and sg5(KO2) were selected for further CRISPR knockout experiments.
E The effects of DGUOK depletion (KO2) on H1650 cell proliferation.
F Representative bioluminescence image showing the tumor growth and progression of luciferase-labeled, control, and DGUOK KO2 LLC cells when orthotopically implanted into the left lung of Albino BL6 mice.
G The quantitation of bioluminescence imaging results in (F). Data are shown as mean ± SD (n = 10 mice per group).

© 2019 The Authors

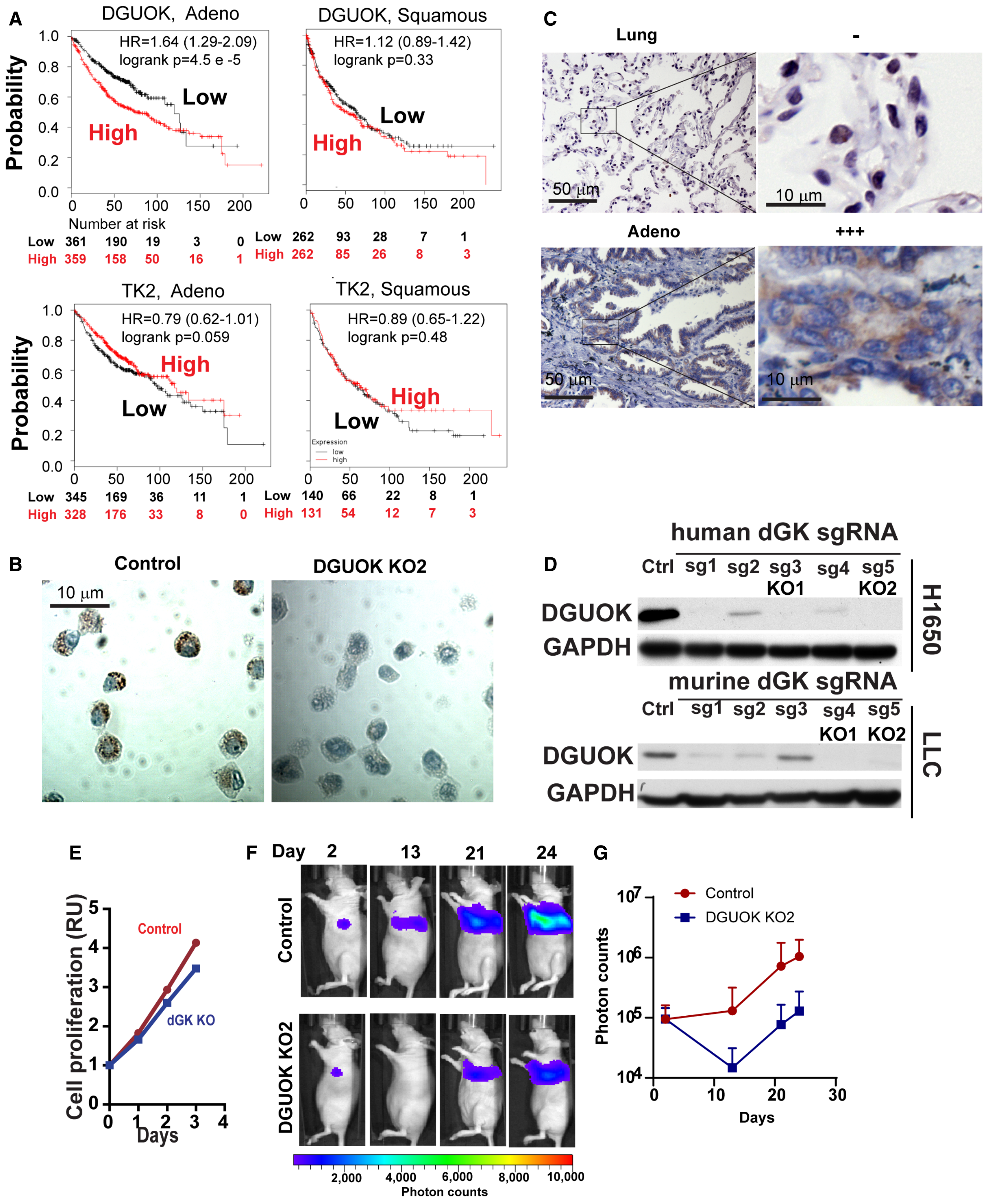

Figure EV1.

 © 2019 The Authors

Figure EV2. The effect of DGUOK KO on CSC population.

A Negative control for the ALDEFLUOR assay data in Fig 2A. DEAB (diethylaminobenzaldehyde), an aldehyde dehydrogenase inhibitor, was used to inhibit ALDH in lung adenocarcinoma cells.

B ALDEFLUOR and anti-CD166 antibody staining were employed to determine the effect of DGUOK KO on $CD166^+$/$ALDH^+$ population in H1650 cells.

C Flow cytometric assay data indicating that all the H1650 cells were positive for CD49f expression.

D Quantitation of the expression levels in control and DGUOK KO2 H1650 cells in (C).

E The effect of DGUOK KO on the "side population (SP)" cells in H1650 and A549 cells, as determined by Hoechst blue and Hoechst red double staining. The SP cells disappeared when cells were treated with fumitremorgin C (FTC), an inhibitor for ABCG2.

F The expression levels of DGUOK in H1650, A549, and PDCs.

G The effects of DGUOK KO on tumor sphere formation in PDCs. Data are shown as mean ± SD from three technical replicates (n = 3). P values were determined by two-tailed, two-sample Student's t-test.

© 2019 The Authors

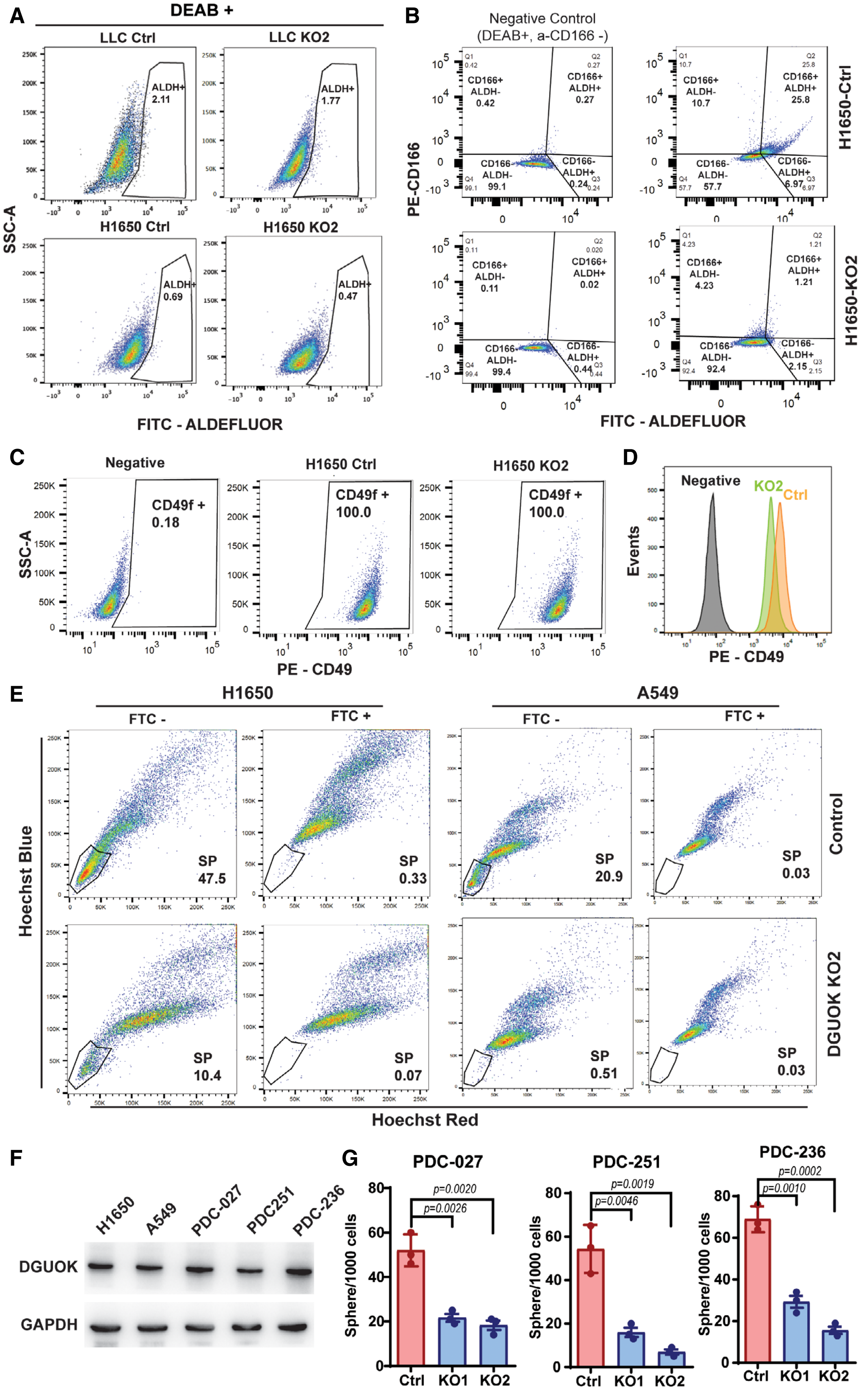

Figure EV2.

 © 2019 The Authors

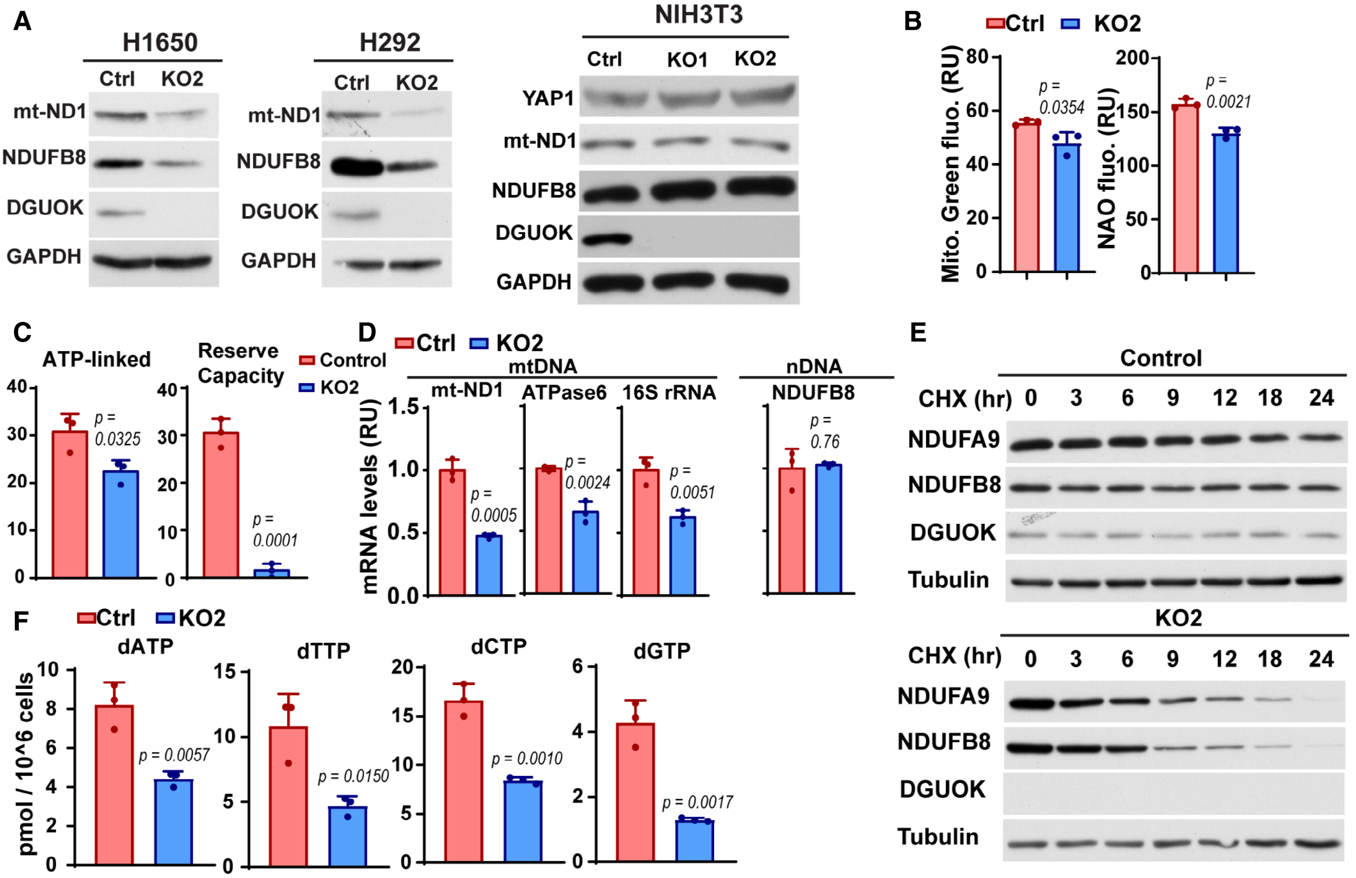

Figure EV3. The effect of DGUOK depletion on the expression of mitochondrial respiratory complex I.

A The effects of DGUOK depletion on the expression levels of complex I proteins in H1650, H292, and NIH3T3 cells.

B The effects of DGUOK depletion on mitochondrial mass, as measured using MitoTracker Green and NAO staining.

C Quantification of the effects of DGUOK depletion on ATP-linked OCR and reserve capacity OCR in H1640 cells.

D The effects of DGUOK depletion on the levels of mtDNA mRNA and nuclear DNA-encoded complex I subunits.

E The protein stabilities of two nuclear DNA-encoded complex I subunits (NDUFA9 and NDUFB8) in controls or DGUOK KO H1650 cells. Cells were treated with 50 µg/ml cycloheximide (CHX) for indicated time to block protein translation, and the levels of remaining proteins were detected by Western blotting.

F The effects of DGUOK KO on cellular dNTP levels in H1650 cells.

Data information: Data are shown as mean ± SD. (B–D, F) $n = 3$ independent replicates per group. P values were determined by two-tailed, two-sample Student's t-test. All experiments were repeated three times using three biological replicates with similar results.

© 2019 The Authors

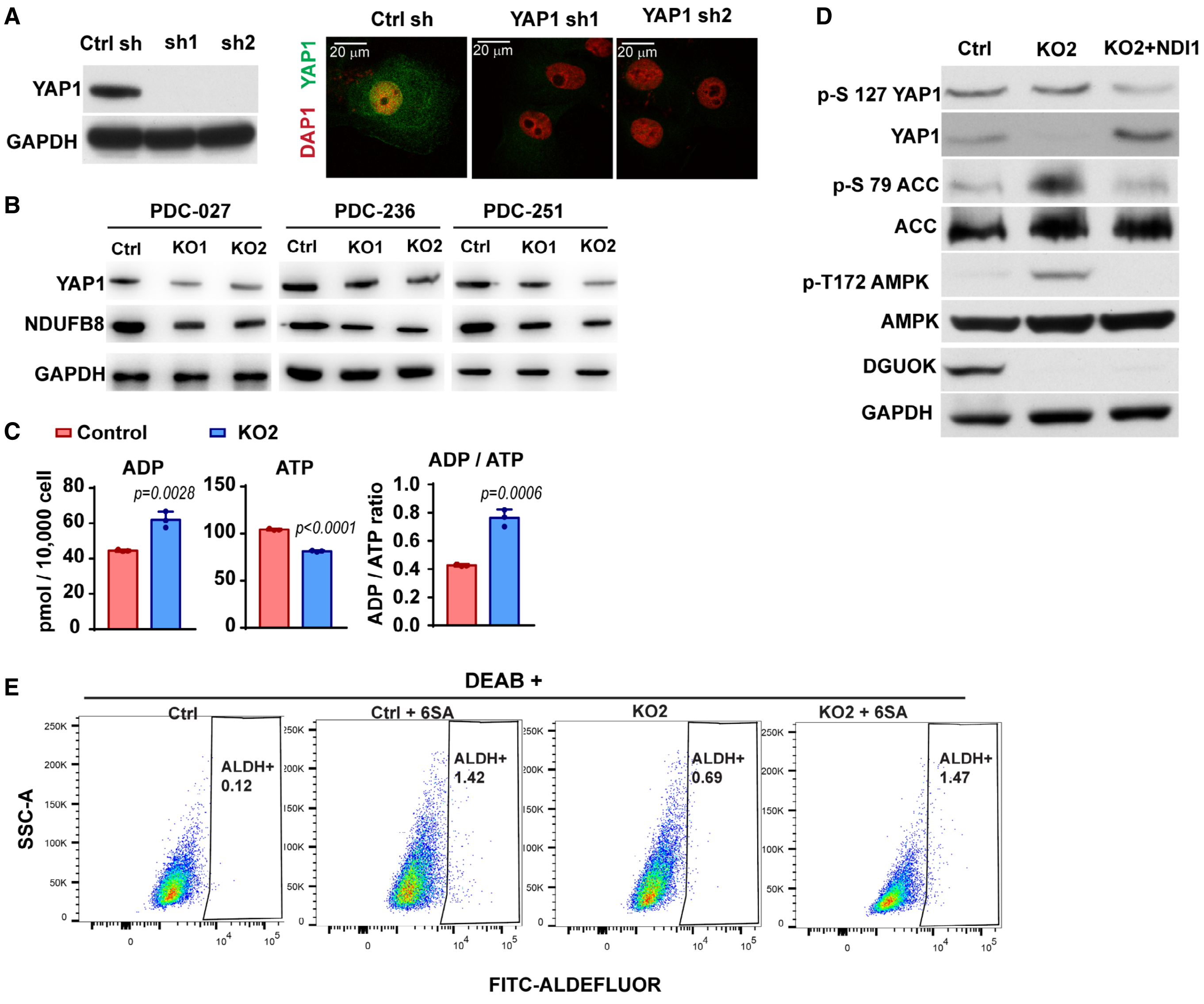

Figure EV4. The regulation of AMPK-YAP1 signaling by DGUOK.

A The effects of YAP1 shRNA on YAP1 protein expression (left panel) and YAP1 immunofluorescence staining (right panel) in H1650 cells. The depletion of YAP1 by two independent shRNA effectively abrogates the immunofluorescence staining by YAP1 antibody, confirming the specificity of the antibody.

B The effect of DGUOK KO on the expression levels of YAP1 and NDUFB8 in three PDCs.

C The levels of ADP, ATP, and ADP/ATP ratios in controls and DGUOK KO2 H1650 cells. $n = 3$ independent replicates per group. P values were determined by two-tailed, two-sample Student's t-test.

D The effect of DGUOK KO and NDI1 ectopic expression on the levels of p-S127 YAP1, YAP1, p-S79 ACC, p-T172 AMPK in A549 cells.

E Negative control for ALDEFLUOR flow cytometric assay in Fig 3L. DEAB (diethylaminobenzaldehyde), an aldehyde dehydrogenase inhibitor, was used to inhibit ALDH in lung adenocarcinoma cells.

© 2019 The Authors

Figure EV5. The role of mitochondrial OXPHOS in DGUOK-mediated regulation of CSC self-renewal.

A Negative control for ALDEFLUOR flow cytometric assay in Fig 6A.

B Negative control for ALDEFLUOR flow cytometric assay in Fig 6B. DEAB (diethylaminobenzaldehyde), an aldehyde dehydrogenase inhibitor, was used to inhibit ALDH in lung adenocarcinoma cells.

C The ectopic expression of yeast NDI1 was able to restore "side population" in DGUOK KO lung cancer cells.

D, E Tumor weight measurement for harvested tumors in Fig 6H and I, respectively. *P* values were determined by two-tailed, two-sample *t*-test. Data are shown as mean ± SD (n = 8 tumors per group).

© 2019 The Authors

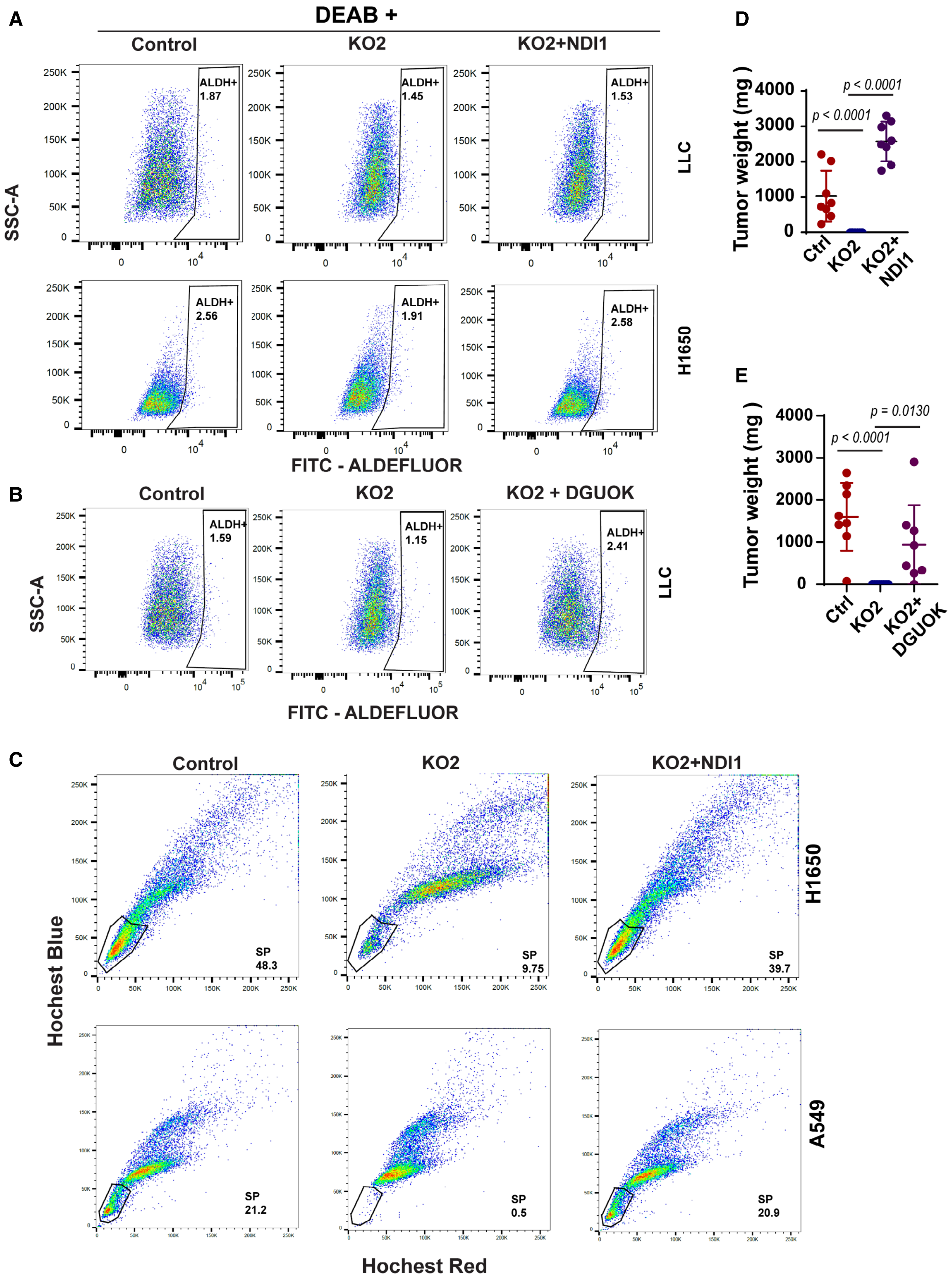

Figure EV5.

© 2019 The Authors
