# Supplementary material for: The mitochondrial deoxyguanosine kinase is required for cancer cell stemness in lung adenocarcinoma
Source: EMBO Mol Med. 2019 Oct 21;11(12):e10849. doi: 10.15252/emmm.201910849 (PMC6895611; doi:10.15252/emmm.201910849)
Supplement: Supplementary file 3 — Table EV1 [file EMMM-11-e10849-s003.docx]

| **Table EV1. Association between DGUOK expression and clinicopathological features of patients with lung adenocarcinoma.** | | | |  |  |  |
| --- | --- | --- | --- | --- | --- | --- |
|  | | | | |  |  |
|  |  | DGUOK expression | |  |  |  |
|  | Total | Low (-/+) | High (++/+++) |  |  |  |
|  | (n = 113) | (n = 71) | (n = 42) | c2 | *P* value |  |
| Gender |  |  |  | 0.951 | 0.430 |  |
| Male | 47 | 32 | 15 |  |  |  |
| Female | 66 | 39 | 27 |  |  |  |
| Age(year) |  |  |  | 0.099 | 0.831 |  |
| <65 | 80 | 51 | 29 |  |  |  |
| ≥65 | 33 | 20 | 13 |  |  |  |
| Smoker |  |  |  | 0.585 | 0.558 |  |
| Yes | 62 | 37 | 25 |  |  |  |
| No | 51 | 34 | 17 |  |  |  |
| Tumor size(cm) |  |  |  | 7.593 | 0.007* |  |
| ≤3.5 | 54 | 41 | 13 |  |  |  |
| >3.5 | 59 | 30 | 29 |  |  |  |
| T staging |  |  |  | 4.739 | 0.038* |  |
| T1 -T2a | 76 | 53 | 23 |  |  |  |
| T2b - T4 | 37 | 18 | 19 |  |  |  |
| Nodal involved |  |  |  | 18.662 | <0.001* |  |
| - | 67 | 53 | 14 |  |  |  |
| + | 46 | 18 | 28 |  |  |  |
| TNM staging |  |  |  | 13.856 | <0.001* |  |
| I-IIA | 58 | 46 | 12 |  |  |  |
| IIB-III | 55 | 25 | 30 |  |  |  |
| ^*^*P*<0.05 (Chi-Square Tests) | |  |  |  |  |  |
