# Supplementary material for: The mitochondrial deoxyguanosine kinase is required for cancer cell stemness in lung adenocarcinoma
Source: EMBO Mol Med. 2019 Oct 21;11(12):e10849. doi: 10.15252/emmm.201910849 (PMC6895611; doi:10.15252/emmm.201910849)
Supplement: Supplementary file 4 — Table EV2 [file EMMM-11-e10849-s004.docx]

**Table EV2. Differentially expressed proteins in DGUOK KO H1650 cells as compared with controls**

Fold changes (log2) in two independent experiments are shown. The mitochondrial proteins are highlighted.

|  | **KO1/CTRL 1** | **KO2 /CTRL 2** |
| --- | --- | --- |
| TNK1 | -2.117 | -1.985430621 |
| TRIT1 | -1.99298 | -1.681465937 |
| C11ORF88 | -1.48928 | -1.634019887 |
| TSSC1 | -1.43768 | -1.126750029 |
| GALNT6 | -1.40682 | -0.689811869 |
| SUMF1 | -1.40627 | -1.320638489 |
| CCDC85C | -1.36193 | -1.610261057 |
| SEC24B | -1.34886 | -1.58601395 |
| NDUFB8 | -1.27446 | -1.129840157 |
| HSPA4L | -1.27003 | -1.182997739 |
| TRABD | -1.24295 | -0.732735047 |
| ATP1A2 | -1.0939 | -1.105200598 |
| SRSA | -1.06474 | -0.93499239 |
| UNG | -0.984 | -0.873628655 |
| MYO1D | -0.96798 | -0.85292468 |
| NOS1AP | -0.94142 | -0.871617164 |
| TTC7A | -0.93286 | -0.879010031 |
| LSS | -0.93187 | -0.984306785 |
| CMSS1 | -0.90329 | -0.734494601 |
| NELFA | -0.88602 | -0.631447267 |
| RCAN1 | -0.88411 | -0.693575197 |
| FN3K | -0.86386 | -0.7476164 |
| AKAP9 | -0.86302 | -0.89810183 |
| SQRDL | -0.85637 | -0.974608528 |
| WDFY1 | -0.85421 | -0.783652767 |
| LYN | -0.84083 | -0.788840721 |
| RNF20 | -0.83188 | -0.604413702 |
| PET100 | -0.8271 | -0.671993477 |
| NACA | -0.82688 | -0.78175767 |
| WWOX | -0.81361 | -1.047723323 |
| RNX3 | -0.81205 | -0.735778496 |
| NNMT | -0.81084 | -0.712729794 |
| RPS7 | -0.80001 | -0.823276619 |
| HMGN4 | -0.79267 | -0.543722069 |
| NDUFB10 | -0.78196 | -0.734897529 |
| PITPNM1 | -0.77779 | -0.926029856 |
| CSRP2 | -0.75316 | -0.740104685 |
| ZBTB21 | -0.73751 | -1.025969405 |
| COL17A1 | -0.73713 | -1.143321821 |
| CHAF1B | -0.73032 | -0.660371862 |
| ZMYND11 | -0.72502 | -0.871031412 |
| ZWINT | -0.71889 | -0.615324994 |
| TPM2 | -0.71262 | -0.703429859 |
| LDLR | -0.70824 | -0.634126454 |
| TTP13 | -0.70721 | -1.037858997 |
| GPT2 | -0.70717 | -0.737688822 |
| ANKIB1 | -0.69692 | -0.568747644 |
| NADC | -0.67354 | -0.803760214 |
| HLA-A | -0.67306 | -0.639180631 |
| PDHB | -0.6686 | -0.546819571 |
| IRF9 | -0.66509 | -1.011541626 |
| CHCHD2 | -0.66102 | -1.121567902 |
| TF | -0.65582 | -0.614126006 |
| PLS3 | -0.64826 | -0.591496979 |
| GULP1 | -0.64182 | -0.682486355 |
| PSMD5 | -0.63059 | -0.57614071 |
| CSGALNACT2 | -0.62718 | -2.014904617 |
| PBRM1 | -0.62195 | -0.631338828 |
| B3GNT3 | -0.62065 | -0.567473408 |
| BCS1L | -0.62043 | -0.66434614 |
| LAMB1 | -0.59833 | -0.553222054 |
| CLTA | -0.59822 | -0.835469508 |
| TMEM159 | -0.59746 | -0.888344621 |
| NR2C2 | -0.59669 | -0.544331128 |
| BCR | -0.59277 | -0.770070703 |
| HDHD1 | -0.58752 | -0.563092019 |
| TPM3 | -0.58748 | -0.781053487 |
| AKAP17A | -0.58093 | -0.634500704 |
| RAB7A | -0.56983 | -0.607291771 |
| GDA | -0.56533 | -0.778084852 |
| PRKAR1A | -0.563 | -0.641367097 |
| PRAME | -0.56113 | -0.558672339 |
| PCM1 | -0.55889 | -0.708618537 |
| SLC27A3 | -0.54229 | -0.600967211 |
| RPUSD2 | -0.53755 | -0.691256094 |
| SNIP1 | -0.52874 | -0.764646728 |
| BAI1 | -0.51788 | -0.592087725 |
| BAG2 | -0.51368 | -0.613837689 |
| SAFB | 0.524693 | 0.655900457 |
| PRPF18 | 0.527463 | 0.701130326 |
| USP10 | 0.53171 | 0.566129985 |
| SNX18 | 0.537942 | 0.628904531 |
| C2CD5 | 0.539813 | 0.518043724 |
| TRUB1 | 0.545041 | 0.798294875 |
| SSB | 0.55052 | 0.776851499 |
| DIABLO | 0.557052 | 0.700160914 |
| UPF1 | 0.559201 | 0.596811364 |
| WIZ | 0.565913 | 0.591538801 |
| FNBP1 | 0.571519 | 0.652185273 |
| CCND1 | 0.578111 | 0.654326359 |
| RNMT | 0.579208 | 0.516541847 |
| PCNA | 0.580866 | 0.582220232 |
| XRCC1 | 0.597534 | 0.621179809 |
| NAA35 | 0.599022 | 0.633984991 |
| MRPL16 | 0.605643 | 0.644212571 |
| CTPS2 | 0.614669 | 0.617961372 |
| NGDN | 0.615943 | 0.495872069 |
| SMU1 | 0.616426 | 0.514700535 |
| GM2A | 0.639973 | 0.501361305 |
| MPI | 0.652307 | 0.541276841 |
| TMEM238 | 0.655989 | 0.745581288 |
| CHTF18 | 0.669679 | 0.828862536 |
| PIPSL | 0.671319 | 1.051378524 |
| PRDM10 | 0.678351 | 0.640649876 |
| EPHX2 | 0.682271 | 0.51223455 |
| ARAP1 | 0.684738 | 0.84289057 |
| SETD3 | 0.711384 | 0.558590314 |
| DDX60L | 0.713841 | 0.648354768 |
| AK4 | 0.723699 | 0.653492583 |
| TIM23 | 0.756198 | 0.986932134 |
| RAB11A | 0.75832 | 0.928162221 |
| COL4A2 | 0.794502 | 1.02849331 |
| NIPBL | 0.804704 | 0.937263842 |
| RBM6 | 0.818902 | 1.059202874 |
| C11ORF54 | 0.858842 | 1.544534082 |
| EARS2 | 0.885931 | 0.511218041 |
| RGPD3 | 0.921721 | 1.020398457 |
| CLIP4 | 0.943355 | 1.397577529 |
| SMYD5 | 1.049775 | 0.663207094 |
| ISCA2 | 1.083397 | 1.496376592 |
| RNF220 | 1.214553 | 0.925145561 |
| CTPS1 | 1.254104 | 0.837967203 |
| COX6A1 | 1.281101 | 1.140552835 |
| PRKCDBP | 1.306174 | 1.651620465 |
| IPO8 | 1.332168 | 0.929397087 |
| ZNF280C | 1.457166 | 1.095323007 |
| MMGT1 | 1.503152 | 1.385492377 |
| HSP90ABP4 | 1.881723 | 1.048658624 |
| SENP1 | 2.263049 | 1.829952732 |
| AGTPBP1 | 2.339537 | 1.734354236 |
|  |  |  |
