# Supplementary figures and images for: The mitochondrial deoxyguanosine kinase is required for cancer cell stemness in lung adenocarcinoma
Source: EMBO Mol Med. 2019 Oct 21;11(12):e10849. doi: 10.15252/emmm.201910849 (PMC6895611; doi:10.15252/emmm.201910849)

Source data for Figure 3

Fig3D

MTND1

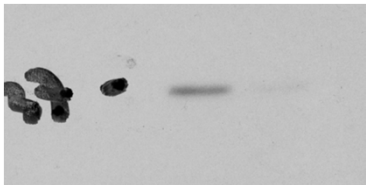

NDUFB8

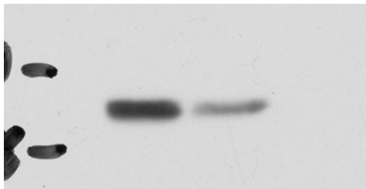

GAPDH

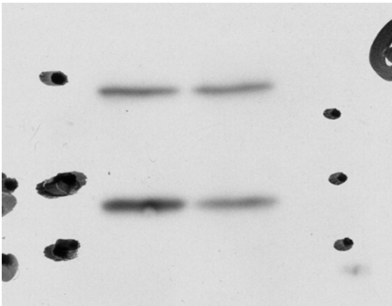

NDUFB10

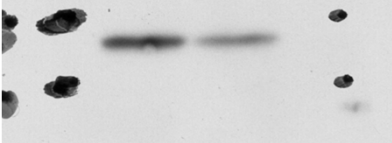

MTCO2

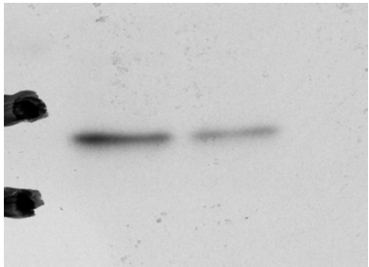

TOM20

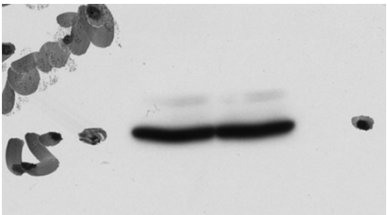

DGUOK

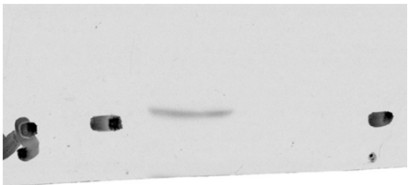

Supplement: Supplementary file 6 — Source Data for Figure 3 [file EMMM-11-e10849-s005.pdf]

Source data for Figure 4

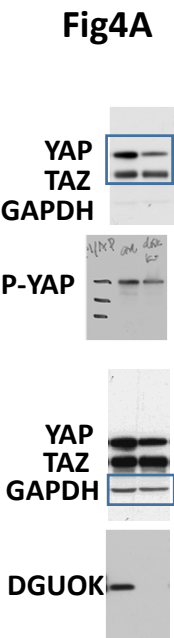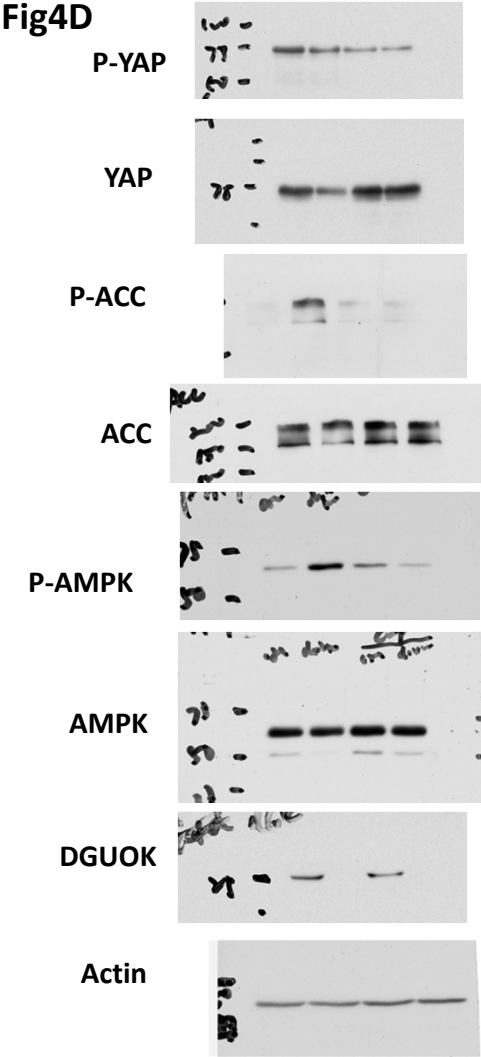

**Fig4E**

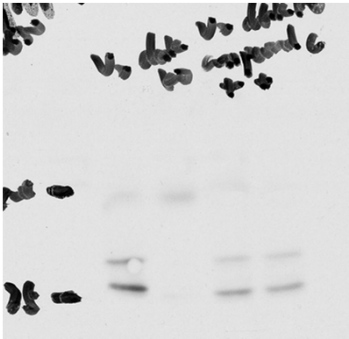

**Fig4J**

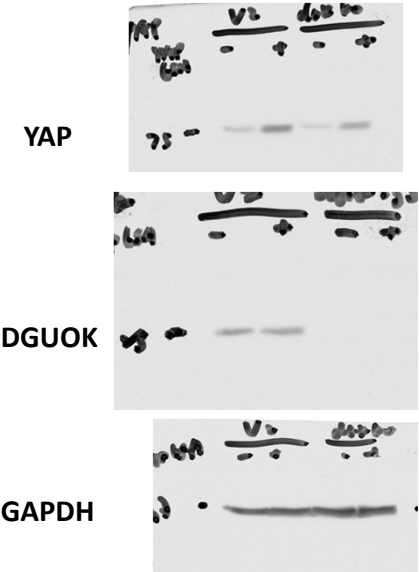

Supplement: Supplementary file 7 — Source Data for Figure 4 [file EMMM-11-e10849-s006.pdf]

Source data for Figure 5

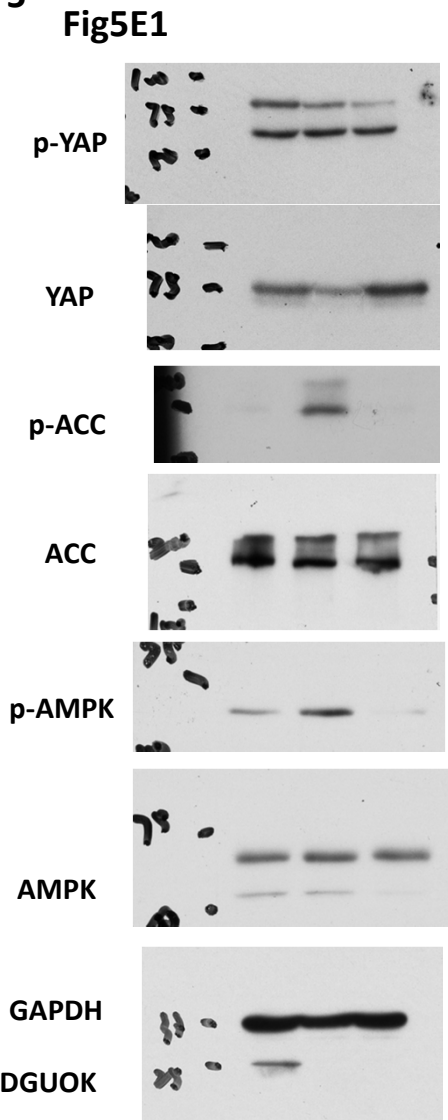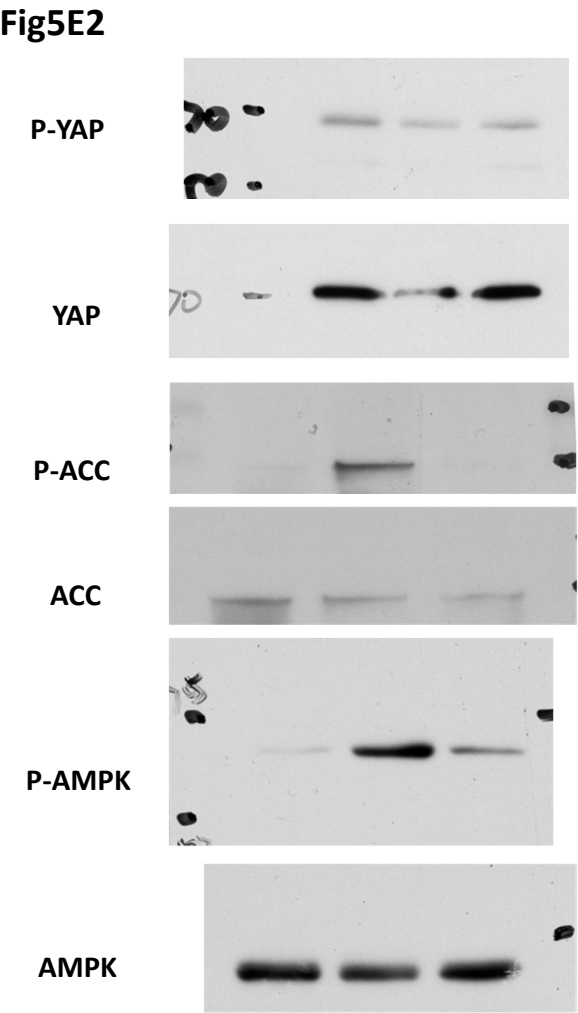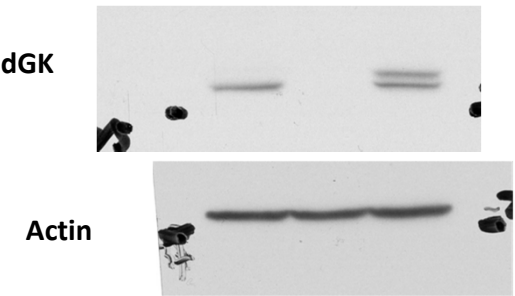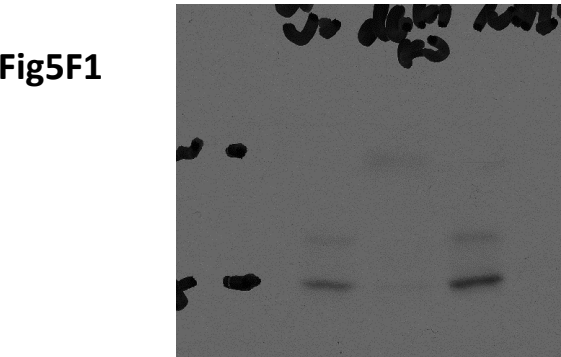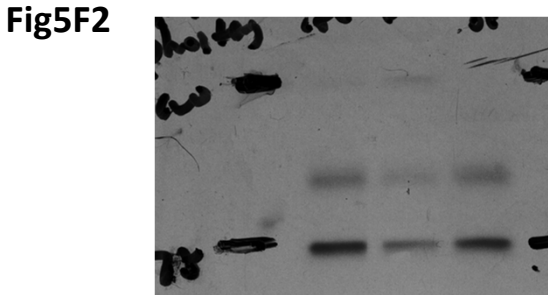

Supplement: Supplementary file 8 — Source Data for Figure 5 [file EMMM-11-e10849-s007.pdf]

Source data for Figure 7

Fig7B

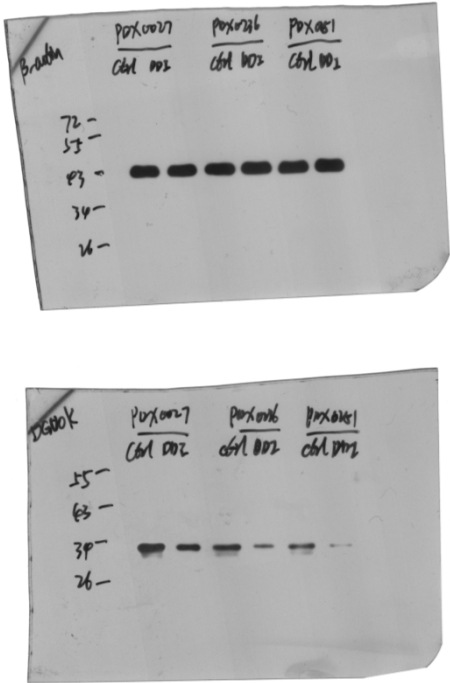

Fig7E

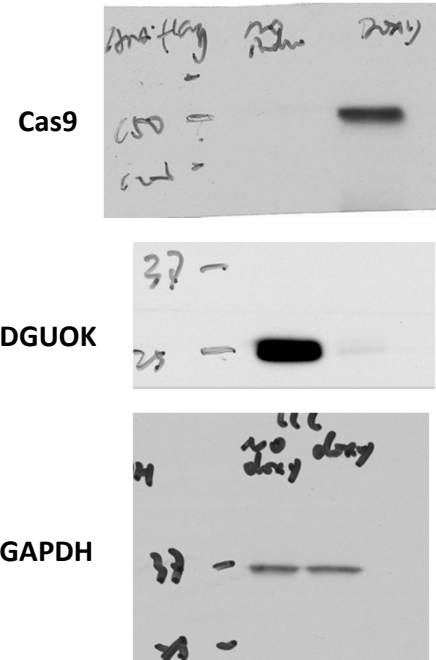

Fig7J

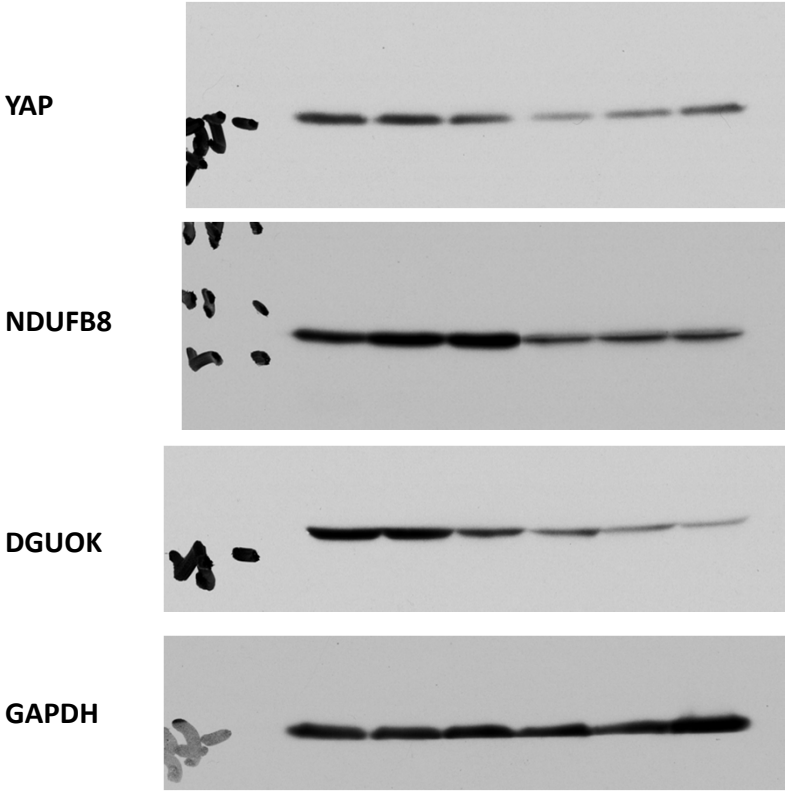

Supplement: Supplementary file 9 — Source Data for Figure 7 [file EMMM-11-e10849-s008.pdf]
